# Laxative use and mortality in patients on haemodialysis: a prospective cohort study

**DOI:** 10.1186/s12882-021-02572-y

**Published:** 2021-11-03

**Authors:** Yu Honda, Seiji Itano, Aiko Kugimiya, Eiji Kubo, Yosuke Yamada, Miho Kimachi, Yugo Shibagaki, Tatsuyoshi Ikenoue

**Affiliations:** 1 Division of Nephrology and Hypertension, Department of Internal Medicine, The Jikei University School of Medicine, Tokyo, Japan; 2 Department of Nephrology and Hypertension, Kawasaki Medical School, Kurashiki, Okayama Japan; 3 The Advanced Emergency Medical Center, Yamanashi Prefectural Central Hospital, Yamanashi, Japan; 4 Department of Nephrology, Ageo Central General Hospital, Saitama, Japan; 5 Department of Nephrology, Shinshu University School of Medicine, Nagano, Japan; 6 Kyoto University Graduate School of Medicine/Human Health Science, 53 Kawahara-cho, Sakyo-ku, Kyoto, 606-8507 Japan; 7 Department of Internal Medicine, Division of Nephrology and Hypertension, St. Marianna University Hospital, Kanagawa, Japan

**Keywords:** Constipation, Laxatives, Mortality, Haemodialysis

## Abstract

**Background:**

Patients on haemodialysis (HD) are often constipated. This study aimed to assess the relationship between constipation and mortality in such patients. In this study, constipation was defined as receiving prescription laxatives, based on the investigation results of “a need to take laxatives is the most common conception of constipation” reported by the World Gastroenterology Organization Global Guidelines.

**Methods:**

This cohort study included 12,217 adult patients on HD enrolled in the Japan-Dialysis Outcomes and Practice Patterns study phases 1 to 5 (1998 to 2015). The participants were grouped into two based on whether they were prescribed laxatives during enrolment at baseline. The primary endpoint was all-cause mortality in 3 years, and the secondary endpoint was cause-specific death. Missing values were imputed using multiple imputation methods. All estimations were calculated using a Cox proportional hazards model with an inverse probability of treatment weighting using the propensity score.

**Results:**

Laxatives were prescribed in 30.5% of the patients, and there were 1240 all-cause deaths. There was a significant association between laxative prescription and all-cause mortality [adjusted hazard ratio (AHR), 1.12; 95% confidence interval (CI): 1.03 to 1.21]. Because the Kaplan-Meier curves of the two groups crossed over, we examined 8345 patients observed for more than 1.5 years. Laxative prescription was significantly associated with all-cause mortality (AHR, 1.35; 95% CI: 1.17 to 1.55). The AHR of infectious death was 1.62 (95% CI: 1.14 to 2.29), and that of cancerous death was 1.60 (95% CI: 1.08 to 2.36). However, cardiovascular death did not show a significant inter-group difference.

**Conclusions:**

Constipation requiring use of laxatives was associated with an increased risk of death in patients on HD. It is important to prevent patients receiving HD from developing constipation and to reduce the number of patients requiring laxatives.

**Supplementary Information:**

The online version contains supplementary material available at 10.1186/s12882-021-02572-y.

## Background

Patients undergoing haemodialysis (HD) are more likely to develop constipation, in which bowel movements are infrequent, and stools are hard to pass [1, 2]. Patients undergoing HD are prone to decreased defecation function due to hardened stool and decreased bowel peristalsis, caused by using phosphate and potassium binders, polypharmacy, fluid deficiency owing to fluid intake restriction or improper dry weight (DW) setting, dietary fibre deficiency owing to limited intake of a plant-based diet for potassium control, and lack of exercise owing to dialysis three times a week for about 4 h each time. Many patients on HD require regular prescriptions of laxatives to treat defecation dysfunction [3, 4]. The proportion of patients on HD who regularly use laxatives is more than five times that of the general population [4].

Previous studies on the general population have reported that constipation is a risk factor for cardiovascular disease (CVD), colorectal cancer, and all-cause mortality [5–8]. Constipation alters the gut microbiota, which has been shown to be associated with atherosclerosis, CVD, cancer, and pneumonia in the general population and in animal models [9–12]. Constipation leads to breathing similar to the Valsalva manoeuvre, which causes increases in the blood pressure (BP) and transient drops in the heart rate; consequently, the BP drops and the heart rate rises, which increases CVD events [13].

On the other hand, in patients undergoing HD, it is not clear whether constipation is associated with mortality. Constipation has been reported to be associated with a reduction in both the physical and mental component score domains of quality of life, which have been shown to be associated with mortality in patients on HD [14, 15]. In addition, constipation can cause faecal impaction, ischaemic enteritis, ileus, urinary tract infection, rectal ulcer, and sigmoid volvulus [16–19], which can be fatal in patients on HD with low reserve for clinical events [20]. Thus, constipation can lead to an increased risk of death in patients undergoing HD.

Therefore, the purpose of our study was to examine whether constipation is associated with an increased risk of mortality in patients undergoing HD. Since the definition of “constipation” differs not only between patients but also between different cultures and regions, the way to define constipation should be carefully considered when studies regarding constipation are conducted. The World Gastroenterology Organization global guideline reported that “a need to take laxatives is the most common conception of constipation,” [21] and many studies that defined constipation as laxative prescription have been carried out [7, 22, 23]. Therefore, we examined the association between mortality and constipation defined by the prescription of laxatives in the current study.

## Materials and methods

### Study population and data sources

This was a prospective cohort study based on the Japan-Dialysis Outcomes and Practice Patterns Study (J-DOPPS). The details of the design of J-DOPPS have been described in previous literature [24, 25]. The patients enrolled in J-DOPPS were randomly selected from 107 dialysis centres in Japan, and the data were collected with patient consent. Patients who had been on dialysis for < 3 months were excluded. Information on demographics, comorbidities, and medications was obtained at the time of study enrolment. The maximum follow-up period for each patient was 3 years. In our study, we used data from phases 1 to 5 (1998 to 2015) of J-DOPPS and analysed outpatient maintenance of patients on HD ≥ 18 years of age.

### Exposure of interest

Constipation, the main exposure factor, was defined as the use of laxatives [21] at the time of J-DOPPS case enrolment, and patients prescribed laxatives at their hospital were considered to be using laxatives. Laxatives were clearly defined according to the following drug classifications: 1) “A06 DRUGS FOR CONSTIPATION” in the *anatomical therapeutic chemical* classification, and 2) The Kampo formulas and crude drugs defined as purgative drugs in the *Kyoto Encyclopaedia of Genes and Genomes* [26, 27]. Laxatives that were not listed in these definitions were defined in discussions with an expert panel.

The list of drugs defined as laxatives in this study was as follows: *dioctyl sodium sulfosuccinate, castor oil, aromatic castor oil, bisacodyl, sennoside, senna, coptis rhizome, senna leaf, rhubarb, magnesium oxide and magnesium sulfate hydrate, sodium picosulfate hydrate, rhubarb, aloe, sodium bicarbonate and anhydrous monobasic sodium phosphate, glycerine, polycarbophil calcium, carmellose sodium, agar, magnesium oxide, magnesium sulfate hydrate, D-sorbitol, lactulose, lactitol hydrate, lactitol hydrate, glycerine, sodium bicarbonate and anhydrous monobasic sodium phosphate, bisacodyl, mosapride citrate hydrate, san’oshashinto, daijokito, shojokito, choijokito, tokakujokito extract, daiobotampito, daiokanzoto extract, keishikashakuyakudaioto, mashiningan, junchoto, oshosan, kyuosan, tsudosan, daikenchuto, and lubiprostone* (Supplementary Table S1; Additional file 1).

### Outcomes

The primary outcome was defined as all-cause death occurring within 3 years from the commencement of observation. The secondary outcomes were cause-specific death rates (infection, malignancy, and CVD) (Supplementary Table S2; Additional file 1).

### Statistical analysis

In this study, we compared all-cause mortality for the first 3 years of observation in two groups with and without laxative prescriptions at the beginning of patient follow-up in each J-DOPPS phase. First, we performed a univariate analysis of the prescription status of laxatives. We then performed a multivariate analysis with the inverse probability of treatment weighting (IPTW) method using the propensity score to adjust for confounding factors [28, 29]. Age, sex, dialysis vintage, smoking status, presence of comorbidities (coronary artery disease, cancer, gastrointestinal bleeding, diabetes, hypertension), 12-Item Short-Form Health Survey (SF-12) composed of mental and physical components, single pool Kt/V, normalised protein catabolic rate, type of vascular access, systolic and diastolic BP before dialysis sessions, medication (potassium and phosphate binders), anticholinergic cognitive burden (ACB) scale [30], white blood cell count, lymphocyte count, haemoglobin, haemoglobin A1c (HbA1c), serum albumin, iron, total iron-binding capacity, ferritin, sodium, potassium, phosphate, calcium, parathyroid hormone, triglyceride, high-density lipoprotein-cholesterol, low-density lipoprotein-cholesterol, and uric acid were used to estimate propensity score. Anticholinergic drugs cause constipation and deteriorate the outcomes and can be one of the confounding factors. The ACB scale contains 99 individual drugs whose anticholinergic effects have been assessed by a multidisciplinary panel based on a systematic literature review and expert opinion, and it is currently the most validated anticholinergic scale [31]. Every observation belongs directly to two clusters at once: the DOPPS phase and facility. Multivariate imputation according to a cross-classified data method was used to impute missing variables [32]. Twenty copies of data were used, each with missing values suitably imputed. We estimated mean hazard ratios (HRs) and adjusted 95% confidence intervals (CIs) within the strata using Cox proportional hazards model, and the results in each stratum were combined using Rubin’s rules [33, 34]. The proportional hazards assumption of Cox regression was tested using the correlation coefficient between transformed survival time and the scaled Schoenfeld residuals.

In the sensitivity analysis, we limited patients to those who had been observable for more than 1.5 years. Additional analysis considering type of laxatives was carried out using multiple propensity score [35]. Considering competing outcomes, deaths caused by reasons we were not interested in were defined as censored in the examination of the association between laxative prescription and cause-specific death rate. Other methodological settings were the same as in the main analysis.

All analyses were performed using R 3.63 (R Foundation for Statistical Computing, Vienna, Austria; www.Rproject.org/) software.

## Results

### Characteristics of study subjects

**Fig. 1:**
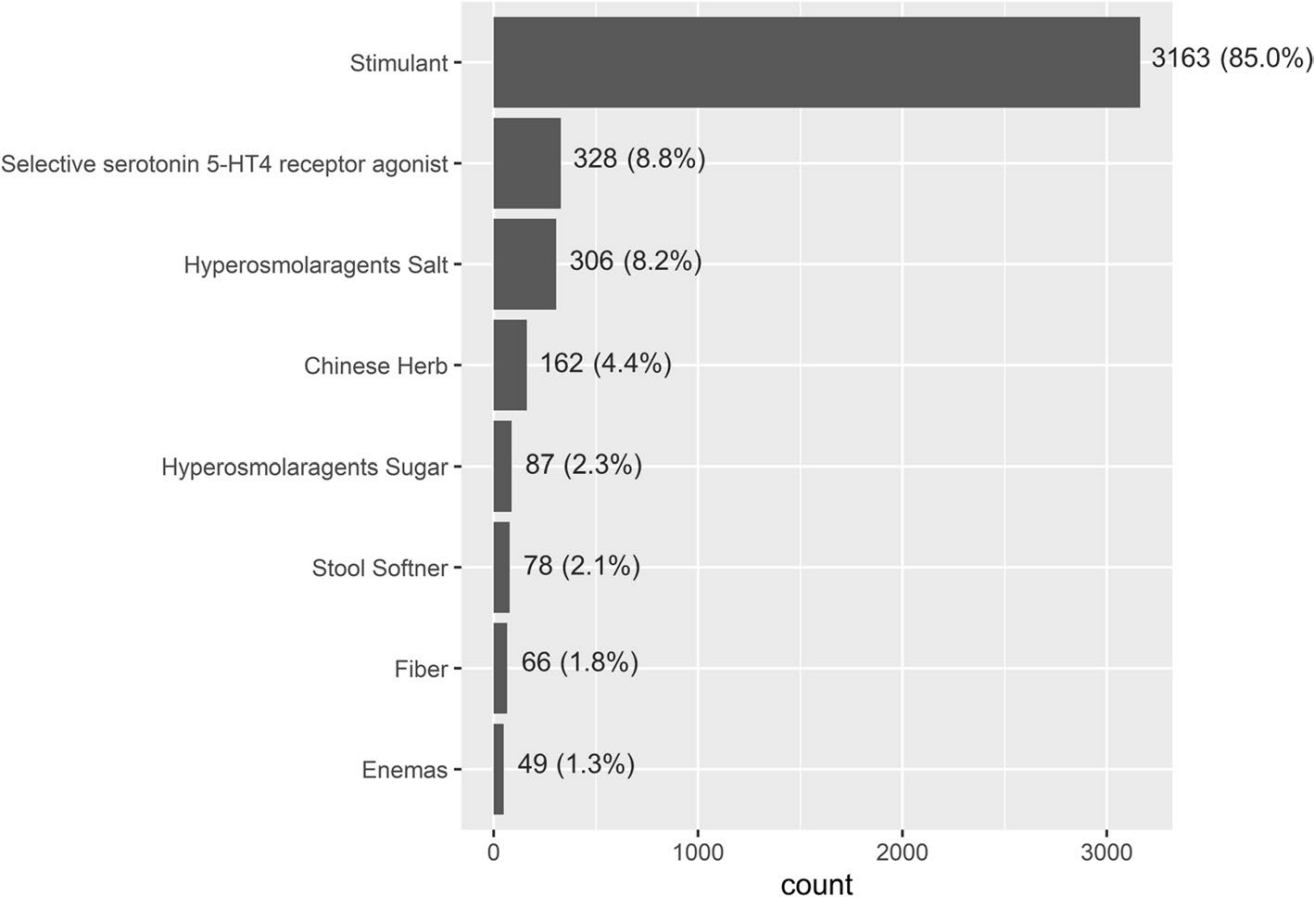
Types of laxatives prescribed for HD patients at enrolment. The number (percentage) of patients who were prescribed laxatives is displayed. See the classification of laxatives in Supplementary Table S1 in Additional file 1. Abbreviations: HT, hydroxytryptamine

A total of 14,212 patients participated in J-DOPPS phases 1 to 5 (Supplementary Fig. S1; Additional file 1). Finally, data from 12,217 patients undergoing HD were used for analysis, excluding 1995 patients with a dialysis history of < 3 months. Patients were divided into two groups: 8496 (69.5%) patients belonged to the no-laxative group and 3721 (30.5%) to the laxative group. In the laxative group, 85.0% of patients orally consumed a stimulant, which was the most common type of laxative, followed by 8.8% who received a selective serotonin 5-HT4 receptor agonist, and 8.2% who received a hyperosmolar agent salt (Fig. 1). The mean age, percentage of men, and dialysis history of the no-laxative and laxative groups at the time of J-DOPPS enrolment were 61.9 vs 64.9 years, 66.5% vs 55.4%, and 6.01 vs 6.77 years, respectively (Table 1). The medical history of the no-laxative and laxative groups included CVD in 26.1 and 31.9%, cancer in 8.4 and 9.8%, and diabetes in 33.8 and 38.3%, respectively. After IPTW was adjusted using propensity score, all covariates were well balanced (i.e., standardised differences were < 0.1).

**Table 1:** Characteristics of the pooled study population of 12,217 persons, according to laxative-use status

Characteristic	No-laxative group *n* = 8496				Laxative group *n* = 3721			Standardised difference		
	Value		Missing		Value		Missing		Before IPTW	After IPTW
Age (years old)	61.9	(13.1)	4	(0.0)	64.9	(11.7)	2	(0.0)	0.226	0.020
Sex (Male)^a^	5642	(66.5)	6	(0.0)	2063	(55.4)	0	(0.0)	−0.116	0.000
Vintage of HD (years)^+^	3.29	[0.52 to 8.92]	60	(0.7)	4.75	[1.25 to 10.03]	32	(0.8)	0.097	0.002
AV Fistula^a^	7171	(90.6)	579	(6.8)	3180	(89.1)	153	(4.1)	−0.012	−0.002
Smoker^a^	1317	(16.4)	476	(5.6)	601	(16.7)	129	(3.4)	0.001	0.000
Body mass index (kg/m^2^)	21.16	(3.31)	1000	(11.7)	20.78	(3.30)	323	(8.6)	−0.102	0.002
Comorbidity										
Cardiovascular disease^a^	2215	(26.1)	0	(0.0)	1186	(31.9)	0	(0.0)	0.056	0.005
Cancer^a^	712	(8.4)	0	(0.0)	364	(9.8)	0	(0.0)	0.006	0.000
Diabetes^a^	2874	(33.8)	0	(0.0)	1425	(38.3)	0	(0.0)	0.045	0.005
Gastrointestinal bleeding^a^	338	(4.0)	0	(0.0)	197	(5.3)	0	(0.0)	0.011	0.000
Hypertension^a^	6103	(71.8)	0	(0.0)	2603	(70)	0	(0.0)	−0.027	−0.003
SF-12										
Mental component summary	45.7	(10.5)	2289	(26.9)	44.2	(10.8)	1130	(30.3)	−0.076	0.001
Physical component summary	42.7	(9.45)	2289	(26.9)	40.1	(9.38)	1130	(30.3)	−0.171	− 0.016
Serum exam										
Kt/V	1.23	(0.3)	1202	(14.1)	1.27	(0.32)	436	(11.7)	0.098	−0.010
Normalised PCR (g/kg/day)	0.95	(0.23)	1202	(14.1)	0.95	(0.21)	436	(11.7)	0.005	−0.001
White blood cells (^a^1000/μl)	6.02	(2.04)	1061	(12.4)	6.01	(1.96)	443	(11.9)	0.015	0.001
Lymphocyte (%)	22.1	(7.84)	4052	(47.6)	22.4	(8.18)	1623	(43.6)	0.044	−0.001
Haemoglobin (g/dL)	10.1	(1.41)	329	(3.8)	9.94	(1.4)	128	(3.4)	−0.123	−0.012
Albumin (mg/dL)	3.71	(0.48)	1011	(11.8)	3.69	(0.44)	481	(12.9)	−0.008	−0.009
Glycohemoglobin (%)	6.08	(1.25)	6334	(74.5)	6.24	(1.31)	2833	(76.1)	0.003	0.005
Total cholesterol (mg/dL)	158.2	(37.3)	1663	(19.5)	162.76	(37.4)	659	(17.7)	0.114	−0.005
LDL-cholesterol (mg/dL)	85.6	(30.2)	5796	(68.2)	89.99	(29.2)	2752	(73.9)	0.082	0.002
Triglyceride (mg/dL)	121.0	(79.9)	2603	(30.6)	118.94	(64.9)	1134	(30.4)	−0.019	−0.009
HDL-cholesterol (mg/dL)	46.8	(16.0)	3387	(39.8)	46.82	(15.6)	1482	(39.8)	−0.012	−0.003
Sodium (mEq/L)	138.9	(3.31)	1712	(20.1)	138.74	(3.29)	1104	(29.6)	−0.024	− 0.016
Potassium (mEq/L)	4.91	(0.79)	225	(2.6)	4.82	(0.79)	81	(2.1)	−0.121	0.006
Calcium (mg/dL)	8.90	(0.96)	598	(7.0)	9.01	(0.93)	198	(5.3)	0.138	0.003
Phosphate (mg/dL)	5.55	(1.51)	285	(3.3)	5.39	(1.55)	104	(2.7)	−0.059	0.001
intact PTH (pg/mL)	189.1	(202.7)	2764	(32.5)	181.98	(252.8)	1290	(34.6)	−0.046	−0.005
Ferritin (ng/mL)	273.3	(470.7)	3045	(35.8)	277.68	(489.2)	204	(5.4)	−0.008	0.002
Iron (μg/dL)	62.1	(30.5)	1855	(21.8)	61.45	(30.5)	712	(19.1)	−0.017	−0.002
Total iron binding capacity (μg/dL)	243.8	(54.6)	4690	(55.2)	243.22	(57.9)	1995	(53.6)	−0.007	−0.004
Uric acid (mg/dL)	7.48	(1.52)	1845	(21.7)	7.54	(1.46)	1162	(31.2)	0.040	−0.010
Blood pressure										
Pre-dialysis DBP (mmHg)	77.8	(13.9)	1551	(18.2)	76.33	(14.0)	1187	(31.9)	−0.086	−0.009
Pre-dialysis SBP (mmHg)	139.5	(24.3)	1612	(18.9)	138.38	(25.0)	1192	(32.0)	−0.005	0.003
Post-dialysis DBP (mmHg)	74.7	(13.7)	1664	(19.5)	72.9	(14.0)	1218	(32.7)	−0.114	−0.010
Post-dialysis SBP (mmHg)	149.6	(23.7)	1524	(17.9)	149.21	(24.5)	1172	(31.4)	−0.042	0.000
Medications										
Potassium binders^a^	1041	(12.3)	0	(0.0)	566	(15.2)	0	(0.0)	0.026	0.001
Number of phosphate binder types	0.69	(0.6)	0	(0.0)	0.76	(0.57)	0	(0.0)	0.143	0.007
ACB scale^a^										
0	2709	(31.9)	0	(0.0)	1162	(31.2)	0	(0.0)	−0.013	−0.003
1	3382	(39.8)			1361	(36.6)			−0.025	0.003
≥ 2	2405	(28.3)			1198	(32.2)			0.036	−0.001

Continuous variables are shown as mean (SD) or median [IQR]

*IPTW* inverse probability of treatment weighting, *HD* haemodialysis, *AV* arteriovenous, *PCR* protein catabolic rate, *SF-12* the 12-item short-form, *DBP* Diastolic blood pressure, *SBP* Systolic blood pressure, *ACB* Anticholinergic Cognitive Burden Scale

^a^ Dichotomous variables were expressed as the number (percentage)

### Association of laxative prescriptions with the primary outcome, death

**Fig. 2:**
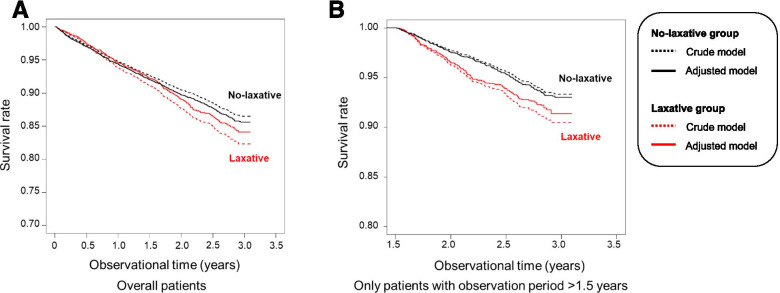
Kaplan-Meier curve comparing the cumulative mortality rates of the no-laxative and laxative groups. **A** Results of all enrolled patients (*N* = 12,217). **B** Results of the patients who could be observed for ≥1.5 years (*N* = 8345). Red line, laxative group; black line, no-laxative group; dash line, crude; solid line, adjusted for potential confounders by inverse probability of treatment weighting method

Figure 2A shows the result of the Kaplan-Meier curve comparing the cumulative mortality rates of the no-laxative and laxative groups in the overall observed period. The median observation period was 769 [interquartile range (IQR): 550 to 1009] days in the no-laxative group and 755 (IQR: 496 to 974) days in the laxative group. During this period, there were 809 (9.7%) and 500 (12.7%) deaths in the no-laxative and laxative groups, respectively. Cox regression analysis showed that the unadjusted HR for the laxative group was 1.35 (95% CI: 1.19 to 1.52), and the adjusted HR (AHR) for potential confounders by the IPTW method was 1.12 (95% CI: 1.04 to 1.22) (Table 2).

**Table 2:** Association of laxative use with the risk of each outcome

Outcomes	Unadjusted HR (95% CI)	Adjusted HR^a^ (95% CI)
**Primary outcome**		
All cause death		
Overall observed patients	1.35 (1.19 to 1.52)	1.12 (1.04 to 1.22)
Patients with observation period > 1.5 years	1.58 (1.28 to 1.96)	1.35 (1.17 to 1.55)
**Secondary outcomes** (in patients with observation period > 1.5 years)		
Infectious death	1.76 (1.05 to 2.94)	1.62 (1.14 to 2.29)
Malignancy death	1.64 (0.91 to 2.94)	1.60 (1.08 to 2.36)
Cardiovascular death	1.38 (1.02 to 1.87)	1.11 (0.91 to 1.36)

^a^Adjusted for age, sex, HD vintage, smoking, comorbidities, blood test, SF-12, spKt/V, normalised PCR, vascular access, blood pressure, potassium binder, phosphate binder, ACB scale by IPTW method

*Abbreviations*: *HR* hazard ratio, *CI* confidence interval

As the Kaplan-Meier curve between groups crossed before 1.5 years of observation and testing rejected the assumption (*p* = 0.029), we could not suspect the proportional hazard assumption between the no-laxative and laxative groups prior to this time. Therefore, we further examined 8345 patients who had been observable for more than 1.5 years (Supplementary Table S3; Additional file 1). Among patients who could be observed for more than 1.5 years, the median follow-up of the no-laxative group was 910 (IQR: 738 to 1039) days, and 254 (4.0%) patients died. In the laxative group, the median observation period was 801 (IQR: 735 to 1081) days, and 173 people (6.0%) died (Fig. 2B). The Cox regression analysis, which focused on patients with an observation period > 1.5 years, showed a statistically significant association with an AHR of 1.35 (95% CI: 1.17 to 1.55) in the laxative group (Table 2).

We also conducted sensitivity analysis to explore the impact of the type of laxatives. Patients in the laxative group were subcategorized into those using stimulant laxative (stimulant laxative group) or those using non-stimulant laxatives (non-stimulant laxative group). The results were similar to the main analysis since all point estimates of AHR were > 1.00 in both groups (Supplementary Table S4: Additional file 1). We could not further subdivide the other types of laxatives because of the small number of patients using each non-stimulant laxative subtype.

### Association between laxative prescription and secondary outcome, cause-specific death rate

We examined the patients who could be observed for ≥1.5 years because of the proportional hazard assumption (Table 2). During the observation period, there were 41 (0.6%) and 29 (1.0%) deaths from infectious diseases, 33 (0.5%) and 24 (0.8%) deaths from cancer, and 135 (2.1%) and 80 (2.8%) deaths from CVDs in the no-laxative and laxative groups, respectively. The Cox regression analysis adjusted for potential confounders by the IPTW method using propensity score and showed that deaths from infection and cancer were significantly associated with laxative prescription [infectious death-AHR 1.62 (95% CI: 1.14 to 2.29) and cancerous death-AHR 1.60 (95% CI: 1.60 to 2.36)]. However, deaths from CVDs were not significant [CVD-AHR 1.11 (95% CI: 0.91 to 1.36)].

## Discussion

In this study, while investigating the relationship between constipation and mortality risk in patients undergoing HD who were administered laxatives for constipation, we observed that the laxative prescription was significantly associated with higher overall mortality risk. Considering each cause of death separately, laxative prescription was significantly associated with infectious disease and malignancy deaths, but not CVD-related death.

This is the first study suggesting that constipation requiring laxative prescription is associated with mortality risk in patients on HD, similar to the results of the general population. A similar tendency was observed in the results of sensitivity analysis wherein the mortality risk in patients prescribed laxative tended to be greater, regardless of the type (stimulant/non-stimulant) of laxative prescribed. The defecation function of patients on HD easily decreases due to iatrogenic factors such as oral administration of phosphorus and potassium adsorbents, exercise and water restriction, insufficient dietary fibre intake due to dietary restrictions, and excessive water removal [1, 36]. Considering the results of this study, to improve the prognosis of patients undergoing HD, it is important for medical caregivers to prevent patients from developing constipation by various methods, such as avoiding the prescription of drugs with the side effect of constipation, avoiding polypharmacy [37], recommending exercise [38, 39], avoiding excessive water removal by the appropriate setting of DW and instruction of appropriate water intake [40], and encouraging fibre consumption from a plant-based diet [41]. On the other hand, while interpreting these results, it should be noted that patients with constipation were defined in this study as those with laxative prescriptions. Therefore, it could not be denied that laxatives themselves might have increased the mortality risk. Hence, it is important to make efforts to reduce the prescription rate of laxatives in patients on HD to reduce the number of patients with constipation by the above considerations.

The following are possible mechanisms to explain the relationship between laxative prescription and increased mortality risk, which is the primary outcome in this study. Constipation leading to laxative prescription can result in a decreased quality of life, which has been implicated in the mortality of patients undergoing HD [14, 15]. Besides, constipation causes the transformation of gut microbiota, which is associated with various fatal diseases [42, 43]. Furthermore, it has been suggested that constipation causes toxic substances in the stool to remain in the intestinal tract, which increases its concentration in the blood and urine [44]. These various mechanisms are thought to function in combination and lead to an increase in the risk of mortality. In this study, the proportional hazards could not be assumed for the association between laxative prescription and outcome incidence during the entire observation period. This is because the Kaplan-Meier curves of the two groups overlapped up to 1.5 years before they separated. The reason for this phenomenon might be that it takes a long time for the harmful effects of constipation, such as the transformation of gut microbiota, to manifest as an increase in mortality.

In the secondary outcomes regarding the cause of death, although malignancy and infectious diseases were associated with laxative prescription, CVD was not significantly associated, unlike the general population. Concerning malignancy and infectious diseases, constipation is associated with a transformation in gut microbiota and intestinal bacterial toxins that can directly or indirectly damage host DNA and cause cancer-enabling mutations [45, 46]. The gut microbiota can promote tumorigenesis by decreasing the production of short-chain fatty acid (SCFA) via fibre metabolism and proinflammatory responses mediated by nuclear factor-κB [46]. SFCA acts on immune cells, such as macrophages and regulatory T cells, and is associated with intestinal immunity and systemic immunity [47]. Carbohydrate fermentation (and thus production of SCFA) and gut microbiota that produce SCFA are reported to be less prominent in patients with constipation [48, 49]. Concerning CVD, it has been indicated that CVD is also related to the transformation of gut microbiota in its onset [50], and many studies have shown a relationship between CVD and laxative prescription or constipation in the general population [13]. Nonetheless, no association between laxative prescription and CVD was found in patients on HD in this study. The possible reasons for this are as follows: first, straining at defecation is one of the causes of CVD [13]. Japanese patients on HD, who have many contacts with doctors [51], are prescribed laxatives more often than is the general population. Therefore, patients on HD receive laxative prescriptions more easily, even if their constipation is mild. Besides, some patients undergoing HD might be suffering from diarrhoea due to the simplistic and aimless administration of laxatives [52]. Therefore, the laxative group in this study might have had relatively few patients who needed to strain during defecation. Secondly, magnesium-containing laxatives might have an influence. Hypomagnesemia is reported to be involved in the onset of CVD. Since the prevalence of hypomagnesemia in patients undergoing HD is higher than that in the general population, administration of magnesium-containing laxatives may have had a stronger effect on suppressing CVD in this cohort [53]. Third, because CVD risk factors in patients on HD are much more diverse than those in the general population, the contribution of constipation to CVD risk may be smaller [54].

This study has several limitations. First, in this study, the cases where constipation was defined by other methods (Bristol scale, Roma IV criteria, defecation frequency, etc.) were not considered. We divided the patients into two groups according to the presence or absence of laxative prescription as a surrogate marker for constipation. Therefore, some patients who did not require laxatives but had impaired defecation function might have been included in the no-laxative group. Hence, the association between constipation and mortality risk could have been underestimated. In the future, verification using another HD patient database in which the actual defecation function is evaluated is required. Second, J-DOPPS does not collect information on over-the-counter laxative drugs. This might have led to information bias. Third, we set exposure using baseline prescription. The laxative prescription may have been started or stopped during the observation period. These changes would have skewed the results to null and would have made the results underestimated. Therefore, they would not affect the conclusions. Fourth, in the sensitivity analysis, both type and amount of laxatives differed between the subgroups using stimulant or non-stimulant laxatives. Therefore, it is unclear whether the statistical difference reflects the impact of type or amount of laxatives. Notably, the amount of laxatives might reflect the severity of constipation. Thus, we need to interpret these results with caution. Lastly, although we adjusted for the influence of potential confounders by some statistical methods in this observational study, there could be a spurious correlation between constipation and mortality [55]; i.e., unmeasured factors such as undiagnosed microscopic cancer, changes in gut microbiota, and progression of micro-arteriosclerosis could have caused constipation; conversely, those unmeasured factors might have caused death from cancers, infection, and CVD. Further validation studies need to be conducted.

## Conclusions

Constipation requiring laxative prescription was associated with higher mortality risk in patients with HD. The cause of death was associated with infectious diseases and malignancy. In the management of patients with HD, medical caregivers should prevent patients from developing constipation and reduce laxative prescriptions.

## Supplementary Information

**Additional file 1: Supplementary Table S1.** Drugs defined as laxatives in this study. **Supplementary Table S2.** Definition of secondary outcomes (cause-specific death) for the current study. **Supplementary Table S3.** Characteristics of study participants observed over 1.5 years. **Supplementary Table S4.** Sensitivity analysis dividing the laxative group into stimulant laxative group or non-stimulant laxative group. **Supplementary Figure S1.** Flow chart of study participants’ selection

## Data Availability

The data that support the findings of this study are available from the Arbor Research Collaborative for Health, but restrictions apply to the availability of these data, which were used under license for the current study, and so are not publicly available. Data are, however, available from the authors (Tatsuyoshi Ikenoue, Ikenoue.tatsuyoshi.4e@kyoto-u.ac.jp) upon reasonable request and with permission of the Arbor Research Collaborative for Health.

## Abbreviations

ACB: Anticholinergic cognitive burden
AHR: Adjusted hazard ratio
AV: Arteriovenous
BP: Blood pressure
CI: Confidence interval
CVD: Cardiovascular disease
DBP: Diastolic blood pressure
DW: Dry weight
HbA1c: Haemoglobin A1c
HD: Haemodialysis
HR: Hazard ratio
HT: Hydroxytryptamine
IPTW: Inverse probability of treatment weighting
IQR: Interquartile range
J-DOPPS: Japan-dialysis outcomes and practice patterns study
PCR: Protein catabolic rate
SBP: Systolic blood pressure
SCFA: Short-chain fatty acids
SF-12: 12-Item Short-Form Health Survey
