# Supplementary material for: Laxative use and mortality in patients on haemodialysis: a prospective cohort study
Source: BMC Nephrol. 2021 Nov 3;22:363. doi: 10.1186/s12882-021-02572-y (PMC8565050; doi:10.1186/s12882-021-02572-y)
Supplement: Supplementary file 1 — Additional file 1: Supplementary Table S1. Drugs defined as laxatives in this study. Supplementary Table S2. Definition of secondary outcomes (cause-specific death) for the current study. Supplementary Table S3. Characteristics of study participants observed over 1.5 years. Supplementary Table S4. Sensitivity analysis dividing the laxative group into stimulant laxative group or non-stimulant laxative group. Supplementary Figure S1. Flow chart of study participants’ selection [file 12882_2021_2572_MOESM1_ESM.docx]

**Additional file 1: Supplementary material**

**Laxatives Use and Mortality in Patients on Haemodialysis: A Prospective Cohort Study**

Yu Honda^1^, Seiji Itano^2^, Aiko Kugimiya^3^, Eiji Kubo^4^, Yosuke Yamada^5^, Miho Kimachi^6^, Yugo Shibagaki^7^, and Tatsuyoshi Ikenoue^6^

^1^Division of Nephrology and Hypertension, Department of Internal Medicine, Jikei University School of Medicine, Tokyo, Japan

^2^Department of Nephrology and Hypertension, Kawasaki Medical School, Kurashiki, Okayama, Japan

^3^The Advanced Emergency Medical Center, Yamanashi Prefectural Central Hospital, Yamanashi, Japan

^4^Department of Nephrology, Ageo Central General Hospital, Saitama, Japan

^5^Department of Nephrology, Shinshu University School of Medicine, Nagano, Japan

^6^Kyoto University Graduate School of Medicine/Human Health Science, Kyoto, Japan

^7^Department of Internal Medicine, Division of Nephrology and Hypertension, St. Marianna University Hospital, Kanagawa, Japan

Corresponding Author:

Tatsuyoshi Ikenoue, MD, PhD

Kyoto University Graduate School of Medicine/Human Health Science

53 Kawahara-cho, Sakyo-ku, Kyoto, 606-8507, Japan

Phone: +075-753-7675; Fax: +075-753-7675

E-mail: ikenoue.tatsuyoshi.4e@kyoto-u.ac.jp

**Supplementary Material**

**Supplementary Table S1.** Drugs defined as laxatives in this study

**Supplementary Table S2.** Definition of secondary outcomes (cause-specific death) for the current study

**Supplementary Table S3.** Characteristics of study participants observed over 1.5 years

**Supplementary Table S4.** Sensitivity analysis dividing the laxative group into stimulant laxative group or non-stimulant laxative group.

**Supplementary Figure S1.** Flow chart of study participants’ selection

Supplementary Table S1. Drugs defined as laxatives in this study

| Type | ATC code | D code | General name |
| --- | --- | --- | --- |
| Stool Softener | A06AA02 | D04406 | Dioctyl sodium sulfosuccinate |
| Lubricant | A06AB05 | D06462 | Castor oil |
|  | A06AB05 | D03418 | Aromatic castor oil |
| Stimulant | A06AB02 | D00245 | Bisacodyl |
|  | A06AB06 | D02171 | Sennoside |
|  | A06AB06 | D08711 | Senna |
|  | A06AB56 | D08711 | Coptis rhizome, senna leaf, rhubarb, magnesium oxide and magnesium sulfate hydrate |
|  | A06AB58 | D01612 | Sodium picosulfate hydrate |
|  | none | none | Rhubarb |
|  | none | none | Aloe |
|  | A06AX02 | none | Sodium bicarbonate and anhydrous monobasic sodium phosphate |
|  | A06AG04(A06AX) | D00028 | Glycerine |
| Bulk-forming laxatives | A06AC08 | D03306 | Polycarbophil calcium |
|  | none | none | Carmelose sodium |
|  | none | none | Agar |
| Hyperosmolar agents/Saline | A06AD02 | D01167 | Magnesium oxide |
|  | A06AD04 | D01108 | Magnesium sulfate hydrate |
| Hyperosmolar agents | A06AD18 | D00096 | D-sorbitol |
|  | A06AD11 | D00352 | Lactulose |
|  | A06AD12 | D08266 D02039 | Lactitol hydrate |
| Enemas | A06AG04(A06AX) | D00028 | Glycerine |
| Suppository | A06AX02 | D04402 | Sodium bicarbonate and anhydrous monobasic sodium phosphate |
|  | A06AB02 | D00245 | Bisacodyl |
| Serotonin 5-HT4 receptor agonists | none | D01994 | Mosapride citrate hydrate |
| Chinese herbal medicine | none | D06967 | San'oshashinto |
|  | none | D07006 | Daijokito |
|  |  | D09119 | Shojokito |
|  |  | D07011 | Choijokito |
|  |  | D07017 | Tokakujokito extract |
|  |  | D07002 | Daiobotampito |
|  |  | D06757 | Daiokanzoto extract |
|  |  | D06942 | Keishikashakuyakudaioto |
|  |  | D07046 | Mashiningan |
|  |  | D06983 | Junchoto |
|  |  | D09050 | Oshosan, Kyuosan |
|  |  | D07016 | Tsudosan |
|  |  | D07003 | Daikenchuto |
| Secretagogues | A06AX03 | D04790 | Lubiprostone |

Supplementary Table S2. Definition of secondary outcomes (cause-specific death) for the current study

| Classification | Cause specific death |
| --- | --- |
| Infection | Lung (pneumonia, flu) |
|  | Abdominal |
|  | Genital urinary tract |
|  | Cardiovascular (Infectious Endocarditis) |
|  | Peritonitis associated with peritoneal dialysis |
|  | Central nervous system |
|  | Others |
|  | Sepsis due to shunt infection |
|  | Peripheral neuropathy, sepsis due to gangrene |
|  | Other sepsis |
| Malignancy | Malignant Tumours |
|  | Cachexia |
| Cardiovascular | Acute myocardial infarction |
|  | Ischemic heart disease |
|  | Valve disease |
|  | Arrhythmia |
|  | Cardiomyopathy |
|  | Pericarditis (including of cardiac tamponade) |
|  | Cardiac arrest of unknown cause |
|  | Bleeding stroke |
|  | Ischemic stroke |
|  | Pulmonary oedema due to fluid retention |
|  | Pulmonary embolism |
|  | Bleeding from an unruptured aneurysm |
|  | Peripheral vascular disease, Sepsis due to gangrene |
|  | Mesenteric infarction, intestinal ischemia |
|  | Calciphylaxis |

Supplementary Table S3. Characteristics of study participants observed over 1.5 years

| Characteristic | | | No-laxative group n=5 760 | | | | | | | | |  | Laxative group n=2 585 | | | | | | | | |  | | Standardised difference | | | | |  |
| --- | --- | --- | --- | --- | --- | --- | --- | --- | --- | --- | --- | --- | --- | --- | --- | --- | --- | --- | --- | --- | --- | --- | --- | --- | --- | --- | --- | --- | --- |
|  |  |  | Value | | | | Missing | | | | |  | Value | | | | Missing | | | | | |  | | before IPTW | | after IPTW | | |
| Age (years old) | | | 60.7 | | (12.7) | | 0 | | (0.0) | | |  | 63.9 | | (11.3) | | 0 | | (0.0) | | | |  | | 0.260 | | 0.027 | | |
| Sex (Male)* | | | 3 784 | | (65.8) | | 0 | | (0.0) | | |  | 1 406 | | (54.4) | | 2 | | (0.0) | | | |  | | -0.118 | | 0.001 | | |
| Vintage of HD (years)^+^ | | | 4.59 | | [1.36 to 10.5] | | 6 | | (0.1) | | |  | 5.41 | | [2.08 to 10.7] | | 0 | | (0.0) | | | |  | | 0.018 | | -0.005 | | |
| AV Fistula* | | | 5 007 | | (92.5) | | 22 | | (0.3) | | |  | 2 238 | | (89.9) | | 8 | | (0.3) | | | |  | | -0.020 | | -0.001 | | |
| Smoker* | | | 908 | | (16.4) | | 346 | | (6.0) | | |  | 423 | | (16.9) | | 95 | | (3.6) | | | |  | | 0.003 | | -0.002 | | |
| Body mass index (kg/m^2^) | | | 21.1 | | (3.24) | | 209 | | (3.6) | | |  | 20.8 | | (3.24) | | 76 | | (2.9) | | | |  | | -0.088 | | 0.005 | | |
| Comorbidity | | |  | |  | |  | |  | | |  |  | |  | |  | |  | | | |  | |  | |  | | |
| Cardiovascular disease* | | | 1 420 | | (24.7) | | 0 | | (0.0) | | |  | 777 | | (30.1) | | 0 | | (0.0) | | | |  | | 0.054 | | 0.003 | | |
| Cancer* | | | 421 | | (7.3) | | 0 | | (0.0) | | |  | 229 | | (8.9) | | 0 | | (0.0) | | | |  | | 0.012 | | 0.000 | | |
| Diabetes* | | | 1 745 | | (30.3) | | 0 | | (0.0) | | |  | 933 | | (36.1) | | 0 | | (0.0) | | | |  | | 0.051 | | 0.008 | | |
| Gastrointestinal bleeding* | | | 199 | | (3.5) | | 0 | | (0.0) | | |  | 107 | | (4.1) | | 0 | | (0.0) | | | |  | | 0.004 | | 0.000 | | |
| Hypertension* | | | 4 102 | | (71.2) | | 0 | | (0.0) | | |  | 1 798 | | (69.6) | | 0 | | (0.0) | | | |  | | -0.024 | | 0.000 | | |
| SF-12 |  | |  | |  | |  | |  | | |  |  | |  | |  | |  | | | |  | |  | |  | | |
| Mental component summary | | | 46.4 | | (10.3) | | 1 167 | | (20.2) | | |  | 44.8 | | (10.6) | | 627 | | (24.2) | | | |  | | -0.084 | | 0.000 | | |
| Physical component summary | | | 43.5 | | (9.11) | | 1 167 | | (20.2) | | |  | 41.0 | | (9.07) | | 627 | | (24.2) | | | |  | | -0.191 | | -0.016 | | |
| Serum exam | | |  | |  | |  | |  | | |  |  | |  | |  | |  | | | |  | |  | |  | | |
| Kt/V | | | 1.27 | | (0.28) | | 510 | | (8.8) | | |  | 1.29 | | (0.31) | | 207 | | (8.0) | | | |  | | 0.054 | | -0.009 | | |
| Normalised PCR (g/kg/day) | | | 0.98 | | (0.22) | | 510 | | (8.8) | | |  | 0.97 | | (0.21) | | 207 | | (8.0) | | | |  | | -0.026 | | -0.003 | | |
| White blood cells (*1000/μL) | | | 5.92 | | (1.89) | | 621 | | (10.7) | | |  | 5.98 | | (1.93) | | 260 | | (10.0) | | | |  | | 0.037 | | 0.001 | | |
| Lymphocyte (%) | | | 22.63 | | (7.62) | | 2 724 | | (47.2) | | |  | 22.77 | | (7.83) | | 1 068 | | (41.3) | | | |  | | 0.042 | | -0.004 | | |
| Haemoglobin (g/dL) | | | 10.28 | | (1.30) | | 149 | | (2.5) | | |  | 10.04 | | (1.33) | | 49 | | (1.8) | | | |  | | -0.176 | | -0.005 | | |
| Albumin (mg/dL) | | | 3.79 | | (0.40) | | 563 | | (9.7) | | |  | 3.75 | | (0.40) | | 284 | | (10.9) | | | |  | | -0.079 | | -0.015 | | |
| Glycohaemoglobin (%) | | | 6.11 | | (1.25) | | 4 428 | | (76.8) | | |  | 6.31 | | (1.29) | | 2 021 | | (78.1) | | | |  | | 0.004 | | 0.005 | | |
| Total cholesterol (mg/dL) | | | 158 | | (35.9) | | 958 | | (16.6) | | |  | 162 | | (36.3) | | 397 | | (15.3) | | | |  | | 0.119 | | -0.008 | | |
| LDL-cholesterol (mg/dL) | | | 85.5 | | (29.6) | | 3 930 | | (68.2) | | |  | 90.5 | | (29.3) | | 1917 | | (74.1) | | | |  | | 0.089 | | 0.002 | | |
| Triglyceride (mg/dL) | | | 121 | | (83.5) | | 1 639 | | (28.4) | | |  | 119 | | (64.2) | | 737 | | (28.5) | | | |  | | -0.016 | | -0.01 | | |
| HDL-cholesterol (mg/dL) | | | 47.3 | | (16.1) | | 2 141 | | (37.1) | | |  | 47.1 | | (15.7) | | 977 | | (37.7) | | | |  | | -0.032 | | -0.008 | | |
| Sodium (mEq/L) | | | 139 | | (3.14) | | 1 247 | | (21.6) | | |  | 138 | | (3.17) | | 823 | | (31.8) | | | |  | | 0.005 | | -0.021 | | |
| Potassium (mEq/L) | | | 5.00 | | (0.75) | | 80 | | (1.3) | | |  | 4.88 | | (0.76) | | 18 | | (0.6) | | | |  | | -0.199 | | 0.007 | | |
| Calcium (mg/dL) | | | 9.02 | | (0.91) | | 313 | | (5.4) | | |  | 9.07 | | (0.92) | | 98 | | (3.7) | | | |  | | 0.086 | | 0.000 | | |
| Phosphate (mg/dL) | | | 5.61 | | (1.44) | | 101 | | (1.7) | | |  | 5.44 | | (1.50) | | 28 | | (1.0) | | | |  | | -0.077 | | -0.001 | | |
| intact PTH (pg/mL) | | | 184 | | (197) | | 1 611 | | (27.9) | | |  | 182. | | (242) | | 808 | | (31.2) | | | |  | | -0.031 | | -0.001 | | |
| Ferritin (ng/mL) | | | 258 | | (451) | | 1 946 | | (33.7) | | |  | 251 | | (439) | | 798 | | (30.8) | | | |  | | -0.021 | | -0.003 | | |
| Iron (μg/dL) | | | 63.7 | | (29.9) | | 1 130 | | (19.6) | | |  | 62.4 | | (30.5) | | 450 | | (17.4) | | | |  | | -0.034 | | -0.01 | | |
| Total iron binding capacity (μg/dL) | | | 247 | | (54.6) | | 3 202 | | (55.5) | | |  | 246 | | (57.3) | | 1 371 | | (53.0) | | | |  | | -0.007 | | -0.003 | | |
| Uric acid (mg/dL) | | | 7.53 | | (1.44) | | 1 282 | | (22.2) | | |  | 7.55 | | (1.38) | | 865 | | (33.4) | | | |  | | 0.034 | | -0.015 | | |
| Blood pressure |  |  | |  | |  | |  | |  |  | | |  | |  | |  | |  |  | | | | |  | |  |  |
| Pre-dialysis DBP (mmHg) | | | 78.1 | | (13.5) | | 254 | | (4.4) | | |  | 77.2 | | (13.6) | | 76 | | (2.9) | | | |  | | -0.052 | | -0.001 | | |
| Pre-dialysis SBP (mmHg) | | | 149. | | (23.1) | | 198 | | (3.4) | | |  | 150 | | (23.8) | | 73 | | (2.8) | | | |  | | 0.031 | | 0.008 | | |
| Post-dialysis DBP (mmHg) | | | 74.7 | | (13.3) | | 185 | | (3.2) | | |  | 73.5 | | (13.7) | | 62 | | (2.3) | | | |  | | -0.074 | | -0.010 | | |
| Post-dialysis SBP (mmHg) | | | 138 | | (23.7) | | 289 | | (5.0) | | |  | 138 | | (24.5) | | 91 | | (3.5) | | | |  | | 0.004 | | 0.000 | | |
| Medications | | |  | |  | |  | |  | | |  |  | |  | |  | |  | | | |  | |  | |  | | |
| Potassium binders* | | | 741 | | (12.9) | | 0 | | (0.0) | | |  | 403 | | (15.6) | | 0 | | (0.0) | | | |  | | 0.026 | | 0.001 | | |
| Number of phosphate binder types | | | 0.75 | | (0.60) | | 0 | | (0.0) | | |  | 0.8 | | (0.56) | | 0 | | (0.0) | | | |  | | 0.143 | | 0.007 | | |
| ACB scale * | 0 | | 1 973 | | (34.3) | | 0 | | (0.0) | | |  | 855 | | (33.1) | | 0 | | (0.0) | | | |  | | -0.013 | | -0.005 | | |
|  | 1 | | 2 241 | | (38.9) | |  | |  | | |  | 948 | | (36.7) | |  | |  | | | |  | | -0.025 | | 0.005 | | |
|  | ≥2 | | 1 546 | | (26.8) | |  | |  | | |  | 782 | | (30.3) | |  | |  | | | |  | | 0.036 | | -0.001 | | |

Continuous variables are shown as mean (SD) and median [IQR]. * Dichotomous variables were expressed as the number (percentage).

IPTW, Inverse probability of treatment weighting; HD, haemodialysis; AV, arteriovenous; PCR, protein catabolic rate; SF-12, the 12-item short-form; DBP, Diastolic blood pressure; SBP, Systolic blood pressure; ACB, Anticholinergic Cognitive Burden Scale

Supplementary Table S4. Sensitivity analysis dividing the laxative group into stimulant laxative group or non-stimulant laxative group.

|  | Adjusted HR* (95% CI) |
| --- | --- |
| **Outcome: all-cause death** |  |
| Overall observed patients |  |
| No-laxative group | Reference |
| Stimulant laxative group | 1.09 (0.99 to 1.19) |
| Non-stimulant laxative group | 1.14 (0.96 to 1.36) |
|  |  |
| Patients with observation period > 1.5 years |  |
| No-laxative group | Reference |
| Stimulant laxative group | 1.43 (1.22 to 1.68) |
| Non-stimulant laxative group | 1.03 (0.73 to 1.45) |

* Inverse probability of treatment weighting method using multiple propensity score was adopted for calculation of adjusted hazard ratio (HR). Adjusted potential confounders were the same as the main analysis. (Table 1) Stimulant laxative group includes patients using stimulant laxatives, and non-stimulant laxative group includes those using non-stimulant laxatives in the laxative group. Abbreviations; CI, confidence interval

**
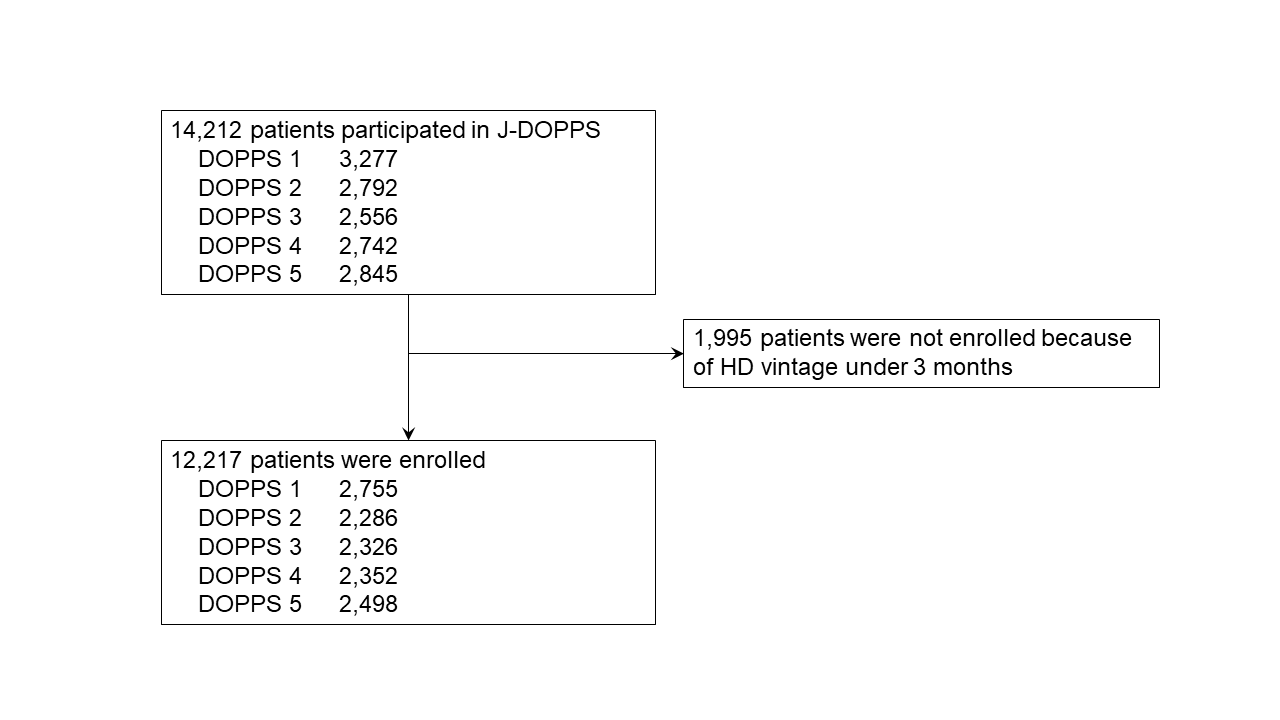
**

Supplementary Figure S1. Flow chart of study participants’ selection

Abbreviations: J-DOPPS, Japan-dialysis outcomes and practice patterns Study; HD, haemodialysis
